# Hypouricemic agents reduce indoxyl sulfate excretion by inhibiting the renal transporters OAT1/3 and ABCG2

**DOI:** 10.1038/s41598-021-86662-9

**Published:** 2021-03-31

**Authors:** Tetsuya Taniguchi, Koichi Omura, Keisuke Motoki, Miku Sakai, Noriko Chikamatsu, Naoki Ashizawa, Tappei Takada, Takashi Iwanaga

**Affiliations:** 1 Research Laboratories 2, Fuji Yakuhin Co., Ltd., 636-1, Iida-Shinden, Nishi-ku, Saitama, Japan; 2 Department of Pharmacy, The University of Tokyo Hospital, Faculty of Medicine, The University of Tokyo, Tokyo, Japan

**Keywords:** Physiology, Nephrology

## Abstract

Indoxyl sulfate (IS) accumulates in the body in chronic kidney disease (CKD). In the renal proximal tubules, IS excretion is mediated by OAT1/3 and ABCG2. These transporters are inhibited by some hypouricemic agents; OATs by probenecid and benzbromarone, ABCG2 by febuxostat and benzbromarone. Thus, we evaluated whether hypouricemic agents including dotinurad, a novel selective urate reabsorption inhibitor with minimal effect on OATs or ABCG2, affect IS clearance in rats. Intact and adenine-induced acute renal failure rats were orally administered hypouricemic agents, and both endogenous IS and exogenously administered stable isotope-labeled d^4^-IS in the plasma and kidney were measured. Our results demonstrated that OATs inhibitors, such as probenecid, suppress IS uptake into the kidney, leading to increased plasma IS concentration, whereas ABCG2 inhibitors, such as febuxostat, cause renal IS accumulation remarkably by suppressing its excretion in intact rats. The effects of these agents were reduced in adenine-induced acute renal failure rats, presumably due to substantial decrease in renal OAT1/3 and ABCG2 expression. Dotinurad did not significantly affected the clearance of IS under both conditions. Therefore, we suggest that hypouricemic agents that do not affect OATs and ABCG2 are effective therapeutic options for the treatment of hyperuricemia complicated by CKD.

## Introduction

Chronic kidney diseases (CKDs) are the pathology of progressive loss of renal function over time based on a gradual decline in the glomerular filtration rate (GFR). The prevalence of CKD is increasing globally; 697.5 million cases were recorded in 2017, with a prevalence rate of 9.1%^1^. Indoxyl sulfate (IS) is a well-known uremic toxin that accumulates under renal impairment and is also involved in the progression of CKD and cardiovascular diseases^2–4^. As for mechanism of action, IS activates NF-κB signaling and increases plasminogen activator inhibitor-1 expression, an associated marker of renal diseases, in human renal proximal tubular epithelial cells^5^. IS also activates NADPH oxidase and induces reactive oxygen species production in human vascular smooth muscle cells and human umbilical vein endothelial cells^6,7^. In rat cardiac cells, IS directly increases collagen synthesis and hypertrophy in cardiac fibroblasts and myocytes, respectively^8^. Furthermore, IS is a ligand of the aryl hydrocarbon receptor, a receptor of dioxin, and altered the transcription of drug metabolism genes^9^.

The kidney has an obligatory role in the excretion of IS from the body, although the involvement of other tissues, intestine and liver, is suggested^10^. For IS excretion from the kidney, the contribution of glomerular filtration is limited by its high plasma protein binding ratio, 93% in humans^11^. Therefore, active transport is important for IS excretion in the renal proximal tubules, and it is well-known that organic anion transporter 1/3 (OAT1/3, also called as SLC22A6/8), which exists in the basolateral membrane, transports IS from blood to epithelial cells^12^. Recently, it has been shown that ATP-binding cassette sub-family G member 2 (ABCG2, also called as breast cancer resistance protein; BCRP), which exists in the brush-border membrane, transports IS from epithelial cells to lumen^10^.

Hyperuricemia is known as a risk factor for CKD onset and progression^13,14^. Although exposure or accumulation of IS in the body is unfavorable for these patients, some hypouricemic agents exhibit potent inhibitory effects against OATs or ABCG2, both of which contribute to excrete IS from the body. In fact, probenecid, a uricosuric agent also known as an OATs inhibitor, decreased IS excretion in the renal proximal tubules and increased IS systemic exposure in rats^15^. Recently, it was reported that febuxostat, a xanthine oxidoreductase inhibitor, and benzbromarone, a uricosuric agent, are potent ABCG2 inhibitors^16^. However, the effect of ABCG2 inhibitors on IS excretion has not been clarified.

Considering these findings, in the present study, we evaluated renal toxicity risk of hypouricemic agents, including dotinurad^17^, a novel selective urate reabsorption inhibitor with minimal effect on OAT1/3 or ABCG2. First, we evaluated hypouricemic agents on the excretion of endogenous IS and exogenously administered stable isotope-labeled d^4^-IS in intact rats. We then evaluated whether the effects of hypouricemic agents persist in adenine-induced acute renal failure rats. To bridge the differences in the results among these rats, the expression of renal transporters was evaluated. We then predict whether these hypouricemic agents interact with OAT1/3 or ABCG2 in clinical situations using in vivo risk factors. Based on the results, the effects of these transporter inhibitors on the kinetics of IS, especially its accumulation in the kidney by an ABCG2 inhibitor will be discussed.

## Results

### Induction of acute renal failure in rats

Several lines of evidence suggest that deposition of 2,8-dihydroxyadenine, a metabolite of adenine, in the renal distal and proximal tubules occurs in adenine-induced kidney injury, and the same phenomenon is also observed by adenine phosphoribosyltransferase deficiency both in mouse and humans^18–20^. In adenine-induced acute renal failure rats, the plasma creatinine levels at one week after the last administration of adenine was 3.4-times higher than that in intact rats (Table 1). Creatinine clearance (CL_CRE_), an index of renal function, was 3.0-times lower than that in intact rats.

**Table 1:** Body weight of and renal functions in intact and adenine-induced acute renal failure rats.

	Intact rats	Adenine-induced acute renal failure rats
Body weight (g)	215 ± 6	182 ± 16**
Plasma creatinine level (mg/dl)	0.29 ± 0.03	1.00 ± 0.12**
CL_CRE_ (ml/min/kg)	8.30 ± 1.53	2.78 ± 0.47**

CL_CRE_: creatinine clearance.

CL_CRE_ is calculated using the following equation: CL_CRE_ = [urine creatinine level (mg/dl) × urine volume (ml/min)] / plasma creatinine level (mg/dl) / body weight (kg).

Each value is presented as mean ± SD of six animals.

**P < 0.01, significantly different from the intact rats according to Student's t-test.

### Hypouricemic agents reduced the excretion of endogenous IS in intact rats

**Figure 1:**
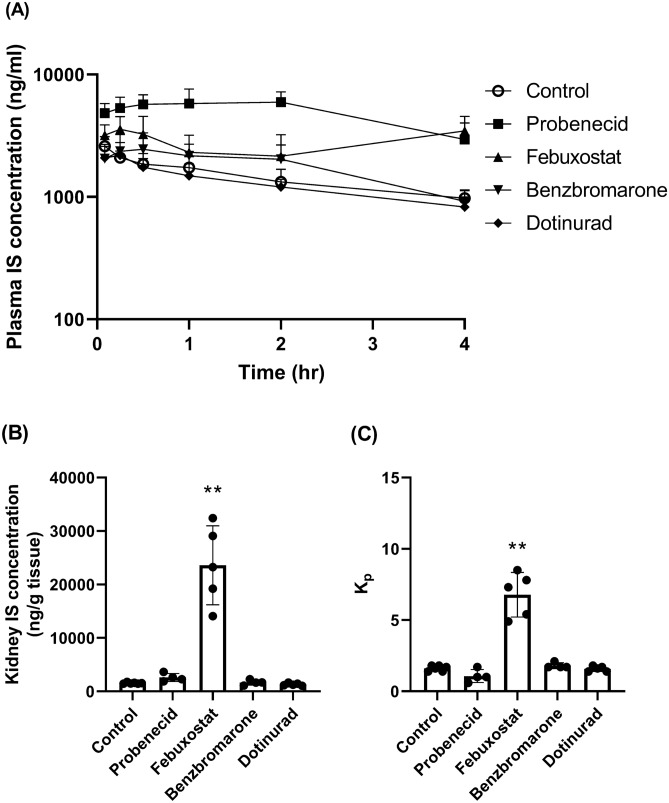
Effect of hypouricemic agents on endogenous IS concentration in the plasma (**A**) and kidney (**B**), and K_p_ (**C**) in intact rats. IS: indoxyl sulfate, K_p_: kidney-to-plasma partition coefficient. Hypouricemic agents or their vehicles as controls were orally administered to Wistar rats fasted for 16 h. After 30 min, d^4^-IS was intravenously administered. Blood samples were collected at 0.083, 0.25, 0.5, 1, 2, and 4 h after d^4^-IS administration. Plasma samples were deproteinated, and the endogenous IS concentration was measured by LC–MS/MS. Each value is presented as mean + SD (**A**) or mean ± SD with individual dots (**B** and **C**) of four to six animals. **: *P* < 0.01, significantly different from the control group according to Dunnett’s multiple comparison test.

The effects of probenecid, febuxostat, benzbromarone, and dotinurad on the plasma concentration of endogenous IS were assessed in intact rats. Probenecid and febuxostat significantly increased the plasma IS concentration (Fig. 1A). The area under the curve from 0 to 4 h (AUC_0-4 hr_) of IS is shown in Table 2. Probenecid and febuxostat increased the AUC_0-4 hr_ by 244% and 85%, respectively, compared with that in control of AUC_0-4 hr_, suggesting IS accumulation in blood. On the contrary, benzbromarone and dotinurad did not have any effects on the plasma IS concentration. It is noteworthy that febuxostat increased the kidney IS concentration and kidney-to-plasma partition coefficients (K_p_) to 15.1- and 4.3-times, respectively, compared with those in control (Fig. 1B and C). Although probenecid tended to increase the kidney IS concentration to 1.7-times, it had no effect on the K_p_ (Table 2). Probenecid and febuxostat decreased the plasma concentration-based renal clearance (CL_R, plasma_) by 73% and 39%, respectively, compared with that in control. Especially, febuxostat decreased the kidney concentration-based renal clearance (CL_R, kidney_) by 93% compared with that of control. These data indicate that plasma and kidney concentration-based parameters are highly sensitive to probenecid and febuxostat, respectively.

**Table 2:** Pharmacokinetic parameters of endogenous IS in intact rats.

Test article	AUC_0-4 hr_ (ng hr/ml)	CL_R, plasma_ (ml/min/kg)	CL_R, kidney_ (ml/min/kg)
Control	5841 ± 1178	6.42 ± 1.14	6.00 ± 1.07
Probenecid	20,067 ± 5249**	1.76 ± 0.56**	3.38 ± 1.08**
Febuxostat	10,781 ± 1717*	3.89 ± 0.55**	0.44 ± 0.06**
Benzbromarone	7260 ± 3096	5.18 ± 1.19	5.62 ± 1.30
Dotinurad	5124 ± 661	7.79 ± 1.04	7.59 ± 1.01

AUC_0-4 hr_: area under the curve from 0 to 4 h, CL_R, plasma_: plasma concentration-based renal clearance, CL_R, kidney_: kidney concentration-based renal clearance.

Data were analyzed using the Phoenix WinNonlin 6.4 software (Certara, L.P., Princeton, NJ) or calculated using the following equations: CL_R, plasma_ = (urine IS concentration × urine volume) / AUC_0-4 hr_, CL_R, kidney_ = (urine IS concentration × urine volume) / (kidney IS concentration at 4 h × 4).

Each value is presented as mean ± SD of four to six animals.

*, **P < 0.05, *P* < 0.01, significantly different from the control group according to Dunnett's multiple comparison test.

### Hypouricemic agents reduced the clearance of d^4^-IS in intact rats

**Figure 2:**
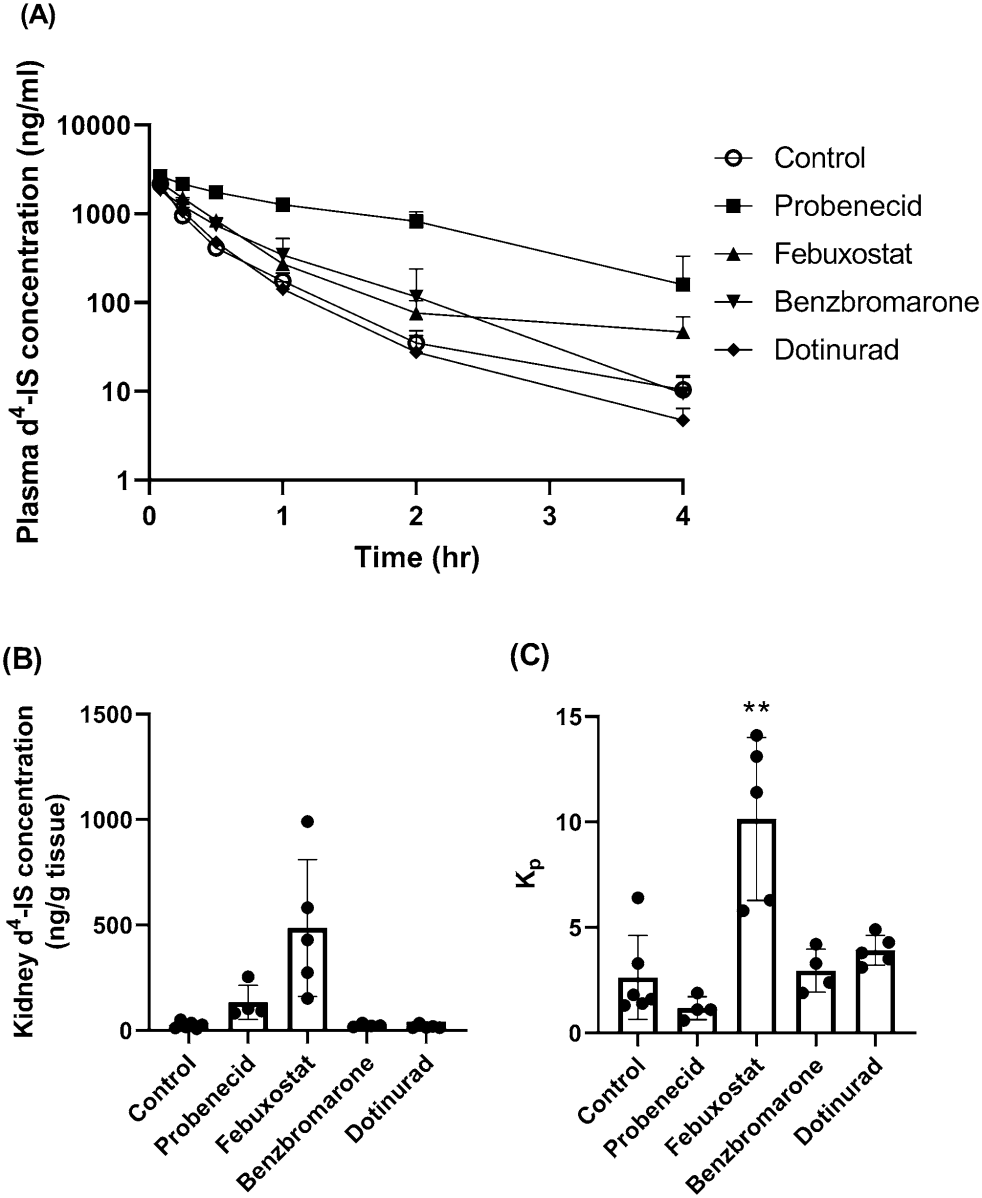
Effect of hypouricemic agents on d^4^-IS concentration in the plasma (**A**) and kidney (**B**), and K_p_ (**C**) in intact rats. IS: indoxyl sulfate, K_p_: kidney-to-plasma partition coefficient. Hypouricemic agents or their vehicles as controls were orally administered to Wistar rats fasted for 16 h. After 30 min, d^4^-IS was intravenously administered. Blood samples were collected at 0.083, 0.25, 0.5, 1, 2, and 4 h after d^4^-IS administration. Plasma samples were deproteinated, and the d^4^-IS concentration was measured by LC–MS/MS. Each value is presented as mean + SD (**A**) or mean ± SD with individual dots (**B** and **C**) of four to six animals. **P < 0.01, significantly different from the control group according to Dunnett’s multiple comparison test.

Exogenously administered IS can be used to accurately evaluate IS excretion, because endogenous IS concentration is affected by two factors; namely, synthesis and excretion. Therefore, the effects of probenecid, febuxostat, benzbromarone, and dotinurad on the plasma and kidney d^4^-IS concentrations were assessed in intact rats (Fig. 2). Probenecid, febuxostat, and benzbromarone decreased the total clearance (CL_tot_) of d^4^-IS by 80%, 44%, and 34%, respectively, compared with that in control (Table 3). Furthermore, probenecid, febuxostat, and benzbromarone decreased the CL_R, plasma_ of d^4^-IS by 78%, 35%, and 43%, respectively, compared with that in control. Of these three agents, probenecid was the most effective on blood concentration-based parameters (CL_tot_ and CL_R, plasma_), indicating that the inhibition of OATs had a large effect on blood concentration-based parameters. In contrast, dotinurad did not have any effect on the CL_tot_ or CL_R, plasma_. It is noteworthy that febuxostat considerably decreased the CL_R, kidney_ of d^4^-IS by 94% compared with that of control, indicating that the inhibition of ABCG2 had a large effect on kidney concentration-based parameters.

**Table 3:** Pharmacokinetic parameters of d^4^-IS in intact rats.

Test article	T_1/2_ (hr)	AUC_0-4 hr_ (ng hr/ml)	CL_tot_ (ml/min/kg)	CL_R, plasma_ (ml/min/kg)	CL_R, kidney_ (ml/min/kg)
Control	0.61 ± 0.07	812 ± 150	6.25 ± 1.02	3.87 ± 0.24	31.4 ± 1.9
Probenecid	1.01 ± 0.45*	3921 ± 632**	1.23 ± 0.26**	0.85 ± 0.08**	6.3 ± 0.6**
Febuxostat	0.70 ± 0.09	1387 ± 125*	3.51 ± 0.36**	2.53 ± 0.08**	1.8 ± 0.1**
Benzbromarone	0.54 ± 0.03	1300 ± 451	4.10 ± 1.09**	2.19 ± 0.35**	28.9 ± 4.6
Dotinurad	0.54 ± 0.09	894 ± 99	5.62 ± 0.63	3.75 ± 0.35	44.9 ± 4.2**

T_1/2_: half-life, AUC_0-4 hr_: area under the curve from 0 to 4 h, CL_tot_: total clearance, CL_R, plasma_: plasma concentration-based renal clearance, CL_R, kidney_: kidney concentration-based renal clearance.

Data were analyzed using Phoenix WinNonlin 6.4 software (Certara, L.P., Princeton, NJ) or calculated using the following equations: CL_R, plasma_ = (urine d^4^-IS concentration × urine volume) / AUC_0-4 hr_, CL_R, kidney_ = (urine d^4^-IS concentration × urine volume) / (kidney d^4^-IS concentration at 4 h × 4).

Each value is presented as mean ± SD of four to six animals.

*, **: *P* < 0.05, *P* < 0.01, significantly different from the control group according to Dunnett's multiple comparison test.

### The effects of hypouricemic agents on the excretion of d^4^-IS in adenine-induced acute renal failure rats

**Figure 3:**
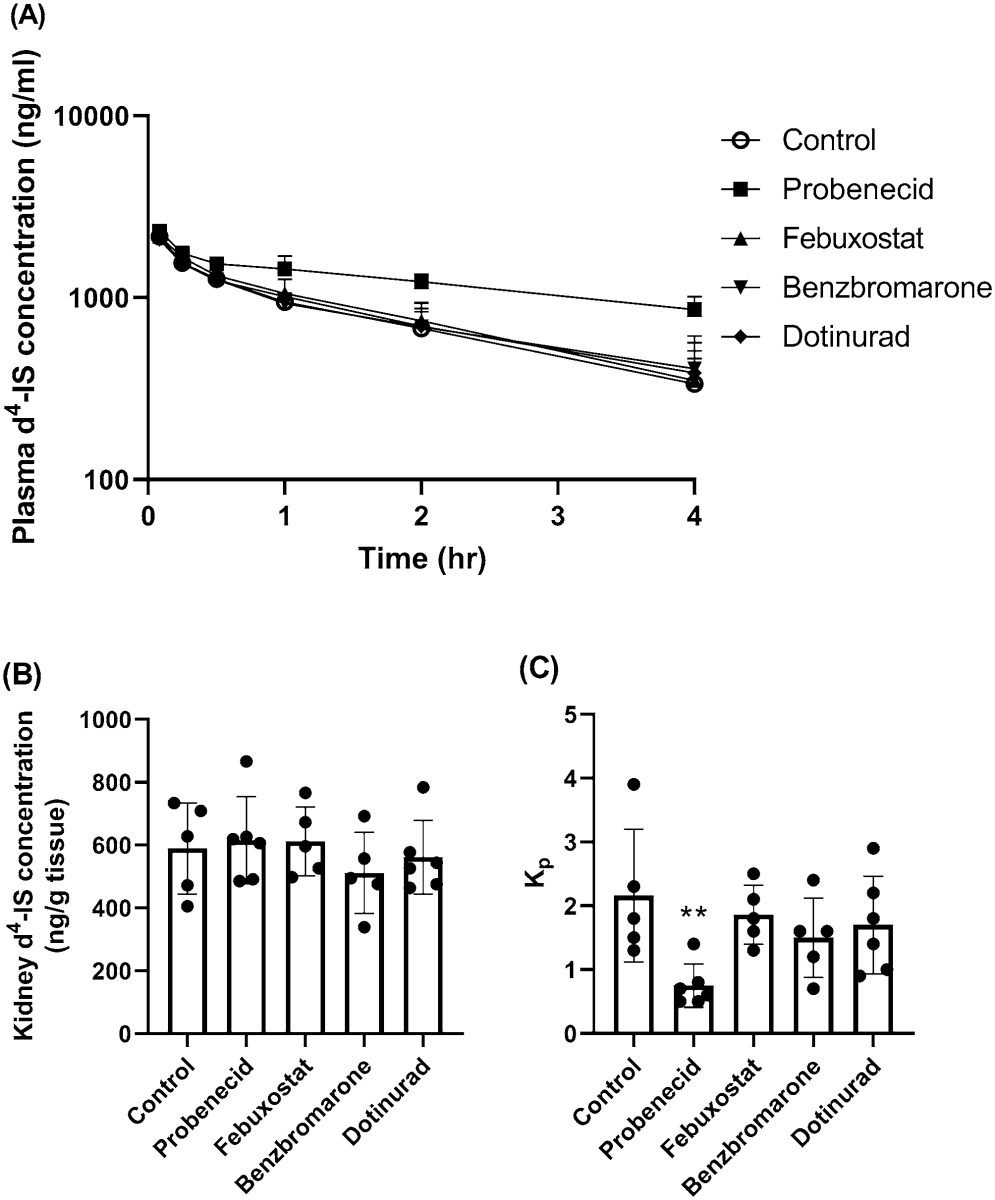
Effect of hypouricemic agents on d^4^-IS concentration in the plasma (**A**) and kidney (**B**), and K_p_ (**C**) in adenine-induced acute renal failure rats. IS: indoxyl sulfate, K_p_: kidney-to-plasma partition coefficient. Hypouricemic agents or their vehicles as controls were orally administered to Wistar rats fasted for 16 h. After 30 min, d^4^-IS was intravenously administered. Blood samples were collected at 0.083, 0.25, 0.5, 1, 2, and 4 h after d^4^-IS administration. Plasma samples were deproteinated, and the d^4^-IS concentrations was measured by LC–MS/MS. Each value is presented as mean + SD (**A**) or mean ± SD with individual dots (**B** and **C**) of five to six animals. **: *P* < 0.01, significantly different from the control group according to Dunnett’s multiple comparison test.

To investigate whether the effects of hypouricemic agents in intact rats were changed in renal impairment, especially under conditions of reduced renal function, the effects of probenecid, febuxostat, benzbromarone, and dotinurad on the plasma concentration of d^4^-IS were assessed in adenine-induced acute renal failure rats. Probenecid showed moderate but significant increase in the plasma concentration of d^4^-IS (Fig. 3 and Table 4). In contrast to the observation in intact rats, febuxostat and benzbromarone did not have any effect on the CL_tot_ of d^4^-IS. Adenine-induced acute renal failure rats show not only a decrease in renal function, but also a considerable loss of IS secretion capacity. Similar to the findings in intact rats, dotinurad did not have any effect on the plasma concentration of d^4^-IS.

**Table 4:** Pharmacokinetic parameters of d^4^-IS in adenine-induced acute renal failure rats.

Test article	T_1/2_ (hr)	AUC_0-4 hr_ (ng hr/ml)	CL_tot_ (ml/min/kg)
Control	2.0 ± 0.8	3234 ± 490	1.26 ± 0.40
Probenecid	5.2 ± 3.6	5112 ± 462**	0.49 ± 0.16**
Febuxostat	1.8 ± 0.2	3475 ± 510	1.18 ± 0.24
Benzbromarone	3.0 ± 1.9	3293 ± 458	1.10 ± 0.44
Dotinurad	2.2 ± 0.6	3349 ± 696	1.19 ± 0.39

T_1/2_: half-life, AUC_0-4 hr_: area under the curve from 0 to 4 h, CL_tot_: total clearance.

Data were analyzed using Phoenix WinNonlin 6.4 software (Certara, L.P., Princeton, NJ).

Each value is presented as mean ± SD of five to six animals.

**P < 0.01, significantly different from the control group according to Dunnett's multiple comparison test.

### Expression of OAT1/3 and ABCG2 in the kidneys of intact and adenine-induced acute renal failure rats

**Figure 4:**
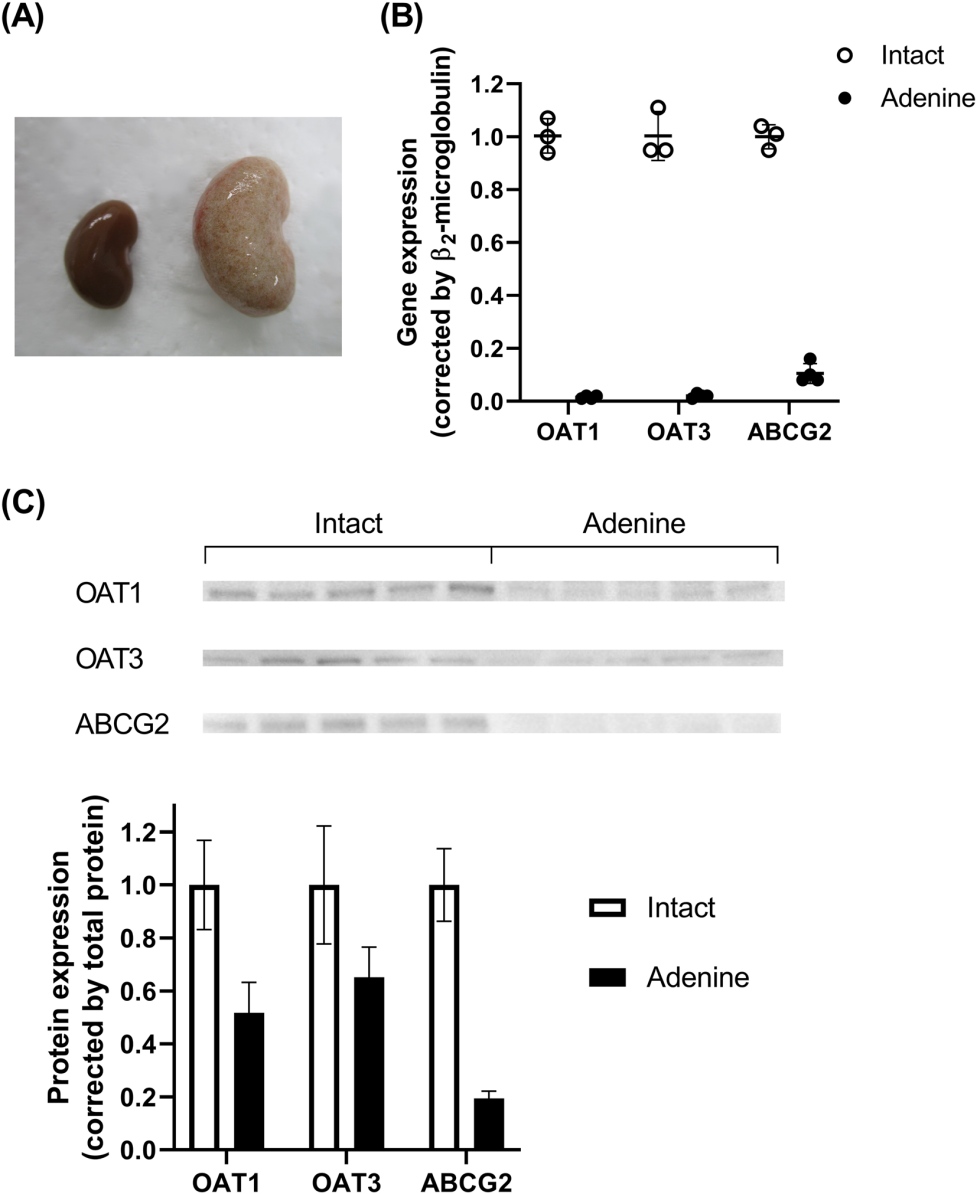
Photograph of the kidneys (**A**), renal expression of OAT1, OAT3, and ABCG2 mRNA (**B)**, and protein (**C**) of intact and adenine-induced acute renal failure rats. The kidneys were obtained from Wistar rats fasted for 16 h. The total RNA and the total membrane protein were extracted from whole kidney. The PCR products of OAT1, OAT3, or ABCG2 gene were normalized to amplified *β*_2_-microglobulin, which was used as the internal reference gene. The chemiluminescence intensity of OAT1, OAT3, or ABCG2 bands in the PVDF membrane was normalized to that of the total protein. The expression of OAT1, OAT3, or ABCG2 in adenine-induced acute renal failure rats was compared with those in intact rats. Each value is presented as mean ± SD with (**B**) or without (**C**) individual dots of three to four animals. Each western blotting image (**C**) is cropped from full-length blots (Supplemental Fig. 1). **P < 0.01, significantly different from the control group according to Student's t-test.

The expression of OAT1/3 and ABCG2 was evaluated, because these transporters are considered to play an important role in IS excretion. In adenine-induced renal failure rats, white colored and larger sized kidneys were observed (Fig. 4A). The mRNA expression of OAT1, OAT3, and ABCG2 normalized to *β*_2_-microgloblin was decreased by 98%, 98%, and 89%, respectively, compared with that in intact rats (Fig. 4B). Furthermore, the protein expression of OAT1, OAT3, and ABCG2 was decreased by 48%, 35%, and 81%, respectively, compared with that in intact rats (Fig. 4C). In adenine-induced acute renal failure rats, the expression of these transporters was substantially decreased, concurrent with a decrease in renal function.

## Discussion

IS is a well-known uremic toxin that accumulates under renal impairment condition and causes toxicity in several tissues. OAT1/3, which exists in the basolateral membrane of renal proximal tubules, transports IS from blood to epithelial cells and ABCG2, which exists in the brush-border membrane of the renal proximal tubules, transports IS from epithelial cells to the lumen, and these transporters are important for the excretion of IS^10,12^. Probenecid, a well-known OATs inhibitor, decreased IS excretion in the renal proximal tubules and increased systemic IS exposure in rats^15^. On the contrary, the effects of ABCG2 inhibitors, such as febuxostat, on IS excretion are not known. In this study, effects of hypouricemic agents including inhibitors of these transporters on IS excretion were examined using both intact and adenine-induced acute renal failure rats, and we tried to predict whether these inhibitions also occurred in clinical situations using the in vivo risk factor.

In the present study, in intact rats, probenecid, febuxostat, and benzbromarone changed endogenous IS and/or d^4^-IS parameters. Probenecid potently changed the plasma concentration-based parameters (AUC_0-4 hr_, CL_tot_ and CL_R, plasma_). In OAT1 knockout mice and OAT3 knockout mice, the plasma concentration of endogenous IS was increased by 9.4- and 3.8-times, respectively, similar to those observed in the present study (1.9–4.5-times)^21,22^. These results indicated that probenecid inhibits IS transport from the blood to the kidney via OAT1/3. On the contrary, the effects of febuxostat on plasma concentration-based parameters were weaker than those of probenecid, whereas febuxostat substantially changed the kidney concentration-based parameters (C_kidney, 4 hr_, K_p_ and CL_R, kidney_). It is noteworthy that the C_kidney, 4 hr_ of endogenous IS in febuxostat-administered intact rats was higher than that in the vehicle-administered adenine-induced acute renal failure rats (Fig. 1 and Supplemental Table 2), indicating that febuxostat largely inhibited IS transport from the epithelial cell to lumen via ABCG2. Benzbromarone seems to inhibit OAT1/3, because it only reduced plasma concentration-based parameters (CL_tot_ and CL_R, plasma_). In contrast, dotinurad did not have any effects on these parameters, and can be considered to hardly inhibit these transporters. These results indicate that the accumulation of IS in the body cannot be determined only from blood concentration-based parameters and that, especially under ABCG2-inhibited conditions, increase in kidney concentration-based parameters occurs prior to increasing blood concentration-based parameters.

In adenine-induced acute renal failure rats, the increasing effects on plasma or kidney IS concentration of probenecid, febuxostat, and benzbromarone were markedly smaller although the plasma concentrations of these drugs were not lower than those in intact rats (Supplemental Fig. 2). This might be caused by the reduced contributions of transporters for IS clearance, because the renal mRNA and protein expression of OAT1/3 and ABCG2 was considerably decreased compared with that in intact rats. Therefore, adenine-induced acute renal failure rats can be regarded as OAT1/3 and ABCG2 down-regulation model. Several studies have reported that the mRNA and protein expression of OATs or ABCG2 was reduced in various renal failure rats^23–25^. There are differences in transporter reduction ratios, although these rats exhibited almost the same (3.7–4.4-fold) increase in blood creatinine levels compared with that in the control rats, and this is similar to the findings of the present study (3.5-fold). Especially, the expression of transporters was substantially affected in the adenine model; 2,8-dihydroxyadenine, a metabolite of adenine deposited in the distal and proximal tubules^18^, may contribute to the phenotype, although the underlying molecular mechanisms remain unknown. In addition, these important results suggest that often-observed decreased clearance of endogenous substances or drugs in CKD conditions may be caused by two factors: renal function impairment, which is usually evaluated by blood creatinine levels or creatinine clearance, and renal tubular injury, including the expression and/or function of transporters. To examine the latter index, endogenous IS measurement is recommended to evaluate the function of OATs or ABCG2. In fact, blood IS concentration is associated with the mortality of patients with acute kidney injury, and this association remained even after adjusting the serum creatinine levels^26^.

We tried to predict whether these hypouricemic agents interact with OAT1/3 or ABCG2 in clinical situations using published data (Supplemental Table 3). In the drug-drug interaction guideline, an in vivo risk factor at > 0.1 means possible interaction in clinical settings^27^. These data indicate that probenecid and benzbromarone have OAT1/3 inhibition risk, and febuxostat and benzbromarone have ABCG2 inhibition risk in clinical practice, with lesser extent for benzbromarone in both cases. In the past, probenecid was used to sustain the serum concentration of penicillin, an OAT1/3 substrate^28^. Recently, it was reported that febuxostat increased the blood concentration of methotrexate and rosuvastatin, which are ABCG2 substrates^29,30^. In the present study, probenecid and benzbromarone should have functioned as OAT1/3 inhibitors and febuxostat as an ABCG2 inhibitor. The less remarkable effect of benzbromarone on ABCG2 compared with febuxostat might be due to its low concentration at the renal proximal tubules, because it has been reported that benzbromarone is excreted mainly via feces^31^.

However, in clinical studies, xanthine oxidoreductase inhibitors, including febuxostat, show renal-protective effects, and reduce urinary albumin-to-creatinine ratio or serum creatinine^32,33^. One of the mechanisms involves the reduction in reactive oxygen species generated by xanthine oxidase (oxidized form of xanthine oxidoreductase)^34^. Recently, an increase in ATP, converted from salvaged hypoxanthine caused by the inhibition of xanthine oxidoreductase, was reported to rescue kidney injury in ischemia reperfusion rats^35^. In the clinical use of febuxostat, deteriorating effect on the kidney caused by the accumulation of IS might be offset by these renoprotective effects. For the accurate evaluation of renal toxicity caused by ABCG2 inhibition, non-renoprotective ABCG2 inhibitors are useful.

In conclusion, probenecid, febuxostat, and benzbromarone affected the excretion of IS in different manners i.e. OATs or ABCG2 inhibition. In particular, febuxostat, as an ABCG2 inhibitor, increased kidney IS concentration, but it cannot be presumed only from blood IS concentration. When treating patients with hyperuricemia who want to avoid accumulation of IS in the body, a hypouricemic agents that do not inhibit OATs or ABCG2, such as dotinurad, are desirable.

## Materials and methods

### Drugs and materials

Probenecid, febuxostat, and benzbromarone were purchased from Tokyo Chemical Industry Co., Ltd. (Tokyo, Japan). Dotinurad was synthesized by Fuji Yakuhin Co., Ltd. (Saitama, Japan). IS potassium salt was purchased from Nacalai Tesque, Inc. (Kyoto, Japan). d^4^-IS potassium salt was purchased from Toronto Research Chemicals, Inc. (Toronto, Canada). Methyl cellulose 400 (MC) was purchased from Wako Pure Chemical Industries, Ltd. (Osaka, Japan). The TaqMan Fast Universal PCR Master Mix was purchased from Thermo Fisher Scientific, Inc. (Waltham, MA). SsoAdvanced Universal SYBR Green Supermix, Precision Plus Protein WesternC Standards, Precision StrepTactin HRP-conjugate, and Clarity Western ECL Substrate were purchased from Bio-Rad Laboratories, Inc. (Hercules, CA). Tris-SDS sample buffer was purchased from Kishida Chemical Co., Ltd. (Osaka, Japan). Primers and antibodies are described in Supplemental Table 4. The other drugs used in the present study are commercially available.

### Animals and housing

To evaluate the inhibitory effects on urate secretion transporters, 7-week-old male Wistar rats bred in Japan SLC, Inc. (Shizuoka, Japan) were used. The rats were housed in wire-mesh cages in an air-conditioned animal room with a 12-/12-h LD cycle at a temperature of 22 °C ± 4 °C and a relative humidity of 60% ± 20% as previously described^17^. The rats that did not develop abnormalities after a 1-week acclimatization period were selected for the study. The rats were fed a CE-2 pellet diet (Clea Japan Inc., Tokyo, Japan) and received tap water via automatic stainless steel nozzles ad libitum throughout the study. The study was carried out in accordance with the ARRIVE guidelines and national animal experiment guidelines, and was approved by the Animal Care and Utilization Committee of Fuji Yakuhin Research Laboratories.

### Induction of acute renal failure in rats

Wistar rats were orally administered 600 mg/kg of adenine, once daily for 5 days. One week after the last administration, urine samples were collected for 4 h. Furthermore, blood samples were obtained from the jugular vein under the isoflurane anesthesia. Plasma was obtained by centrifuging the blood samples at 1000 × g for 10 min at 4 °C. Creatinine level in the plasma and urine was measured using the U-3900 spectrophotometer (Hitachi High-Tech Science Corp., Tokyo, Japan) with the L-type Wako Creatinine F kit (Wako Pure Chemical Industries, Ltd., Osaka, Japan). Creatinine clearance (CL_CRE_) was calculated using the following equation: CL_CRE_ = [urine creatinine level (mg/dl) × urine volume (ml/min)] / plasma creatinine level (mg/dl) / body weight (kg). Rats were selected based on their weight and serum creatinine level. Four days later, the rats were selected based on the weight and serum creatinine level, and used in the study. In the intact group, the rats were used 4 days after the urine and blood sampling.

### Concomitant treatment of d^4^-IS and hypouricemic agents in intact and adenine-induced acute renal failure rats

Wistar rats that were fasted for 16 h before oral drug administration received 100 mg/kg of probenecid, 20 mg/kg of febuxostat, 50 mg/kg of benzbromarone, 1.3 mg/kg of dotinurad, and 0.5% MC as the control. Drug doses used in the present study were calculated based on their clinically maximal doses (Supplemental Table 1) and were same as those in our previous study, in which drug-drug interaction via OAT1 or ABCG2 was observed, except for dotinurad^17^. Thirty minutes after drug administration, 0.3 mg/kg d^4^-IS dissolved in saline was intravenously administered via the tail vein to all rats. Blood samples were obtained from the jugular vein at 0.083, 0.25, 0.5, 1, 2, and 4 h after d^4^-IS administration using a heparinized needle and kept on ice, and then the kidney was isolated with the rats under isoflurane anesthesia. Plasma was obtained by centrifuging the blood samples at 1000 × g for 10 min at 4 °C. To prepare the kidney sample, 4-times volume of saline was added to the sample and homogenized.

### Determination of endogenous IS and d^4^-IS concentrations in the plasma and kidney and analysis of pharmacokinetic parameters

Endogenous IS and d^4^-IS were determined in these samples as previously described with slightly modifications^10^. Briefly, the plasma and kidney homogenates were deproteinized using the internal standard containing methanol. These samples were measured using a liquid chromatography-tandem mass spectrometry (LC–MS/MS) systems consisting of a Nexera X2 and a Prominence instrument (Shimadzu Corp., Kyoto, Japan) coupled with QTRAP4500 (AB SCIEX, LLC., Framingham, MA). Pharmacokinetic parameters (AUC_0-4 hr_, C_0_, V_0_, T_1/2_, and CL_tot_) were analyzed using the Phoenix WinNonlin 6.4 software (Certara, L.P., Princeton, NJ). Some pharmacokinetic parameters were calculated using the following equations, in which "IS" means endogenous IS or d^4^-IS. CL_R, plasma_ = (urine IS concentration × urine volume) / AUC_0-4 hr_. CL_R, kidney_ = (urine IS concentration × urine volume) / (kidney IS concentration at 4 h × 4). K_p_ = kidney IS concentration at 4 h / plasma IS concentration at 4 h.

### Quantitative reverse transcription polymerase chain reaction (qRT-PCR)

The total RNA was extracted from homogenized whole kidney samples using the PureLink RNA mini kit (Thermo Fisher Scientific, Inc., Waltham, MA). RNA concentration was determined by measuring the absorbance at a wavelength of 260 nm. Thereafter, 1 μg of the total RNA was used to prepare complementary DNA by reverse transcription using ReverTra Ace qPCR RT Master Mix (Toyobo Co., Ltd., Osaka, Japan). The levels of mRNA coding for OAT1, OAT3, and ABCG2 were measured using the CFX96 Touch real-time PCR detection system (Bio-Rad Laboratories, Inc.). The amplified products of the target gene were normalized to amplified β_2_-microglobulin, which was used as the internal reference gene.

### Western blotting

The total membrane extract of the whole kidney obtained using the Minute Plasma Membrane Protein Isolation Kit (Invent Biotechnologies, Inc. Plymouth, MN). Twenty micrograms of the total membrane extract was incubated for 2 min at 60 °C after adding an equal volume of Tris-SDS sample buffer and then subjected to sodium dodecyl sulphate polyacrylamide gel electrophoresis (SDS-PAGE). After electrophoresis, Miniprotian TGX Stain-free gel (4–15% or 7.5%) was activated by ultraviolet light for 1 min and the total protein was measured using ChemiDoc XRS Plus (Bio-Rad Laboratories, Inc.). Subsequently, the proteins in the gel were transferred onto PVDF membranes using the Trans-Blot Turbo Transfer System (Bio-Rad Laboratories, Inc.). The membrane was blocked with 0.5% skim milk or 5% bovine serum albumin in wash buffer (25 mmol/l tris buffered saline containing 0.1% Tween 20) for 1 h. The OAT1, OAT3, or ABCG2 antibody in wash buffer was added onto the membrane and incubated overnight at 4 °C. After washing, the membrane was incubated for 1 h with HRP-conjugated goat anti-rabbit IgG antibody in wash buffer. After washing, the ECL substrate was added to the membrane and chemiluminescence was detected using ChemiDoc XRS Plus. The molecular weights of OAT1 (63 kDa), OAT3 (65 kDa), and ABCG2 (68 kDa) were determined as previously described using rat kidney^36–38^. The relative amount of proteins and intensity of bands in each lane were determined using Image Lab (Bio-Rad Laboratories, Inc.). The intensity of OAT1, OAT3, or ABCG2 bands was normalized to that of the total protein.

### Statistical analysis

Mean, standard deviation (SD), CL_R, plasma_, CL_R, kidney_, K_p_ and intensity of the target protein to that of the total protein were calculated using Microsoft Excel Office 365 (Microsoft Corporation, Redmond, WA). Differences from the control group were analyzed using Dunnett’s multiple comparison test and differences between intact and adenine-induced acute renal failure rats were analyzed using Student’s t-test at the significance levels of 5% and 1% with JMP 15.1.0 (SAS Institute Inc., Cary, NC).

## Supplementary Information

Supplementary Information
