# Supplementary material for: Hypouricemic agents reduce indoxyl sulfate excretion by inhibiting the renal transporters OAT1/3 and ABCG2
Source: Sci Rep. 2021 Mar 31;11:7232. doi: 10.1038/s41598-021-86662-9 (PMC8012596; doi:10.1038/s41598-021-86662-9)
Supplement: Supplementary file 1 — Supplementary Information [file 41598_2021_86662_MOESM1_ESM.pdf]

Hypouricemic agents reduce indoxyl sulfate excretion by inhibiting the renal transporters OAT1/3 and ABCG2

Tetsuya Taniguchi[1], Koichi Omura[1], Keisuke Motoki[1], Miku Sakai[1], Noriko Chikamatsu[1], Naoki Ashizawa[1], Tappei Takada[2], and Takashi Iwanaga[1]

[1]Research Laboratories 2, Fuji Yakuhin Co., Ltd., Saitama, Japan

[2]Department of Pharmacy, The University of Tokyo Hospital, Faculty of Medicine, The University of Tokyo, Tokyo, Japan

Corresponding author:

T. Taniguchi. Research Laboratories 2, Fuji Yakuhin Co., Ltd., 636-1 Iida-Shinden, Nishi-ku, Saitama City, Saitama, 331-0068, Japan.

Tel: +81-48-620-1611. Fax: +81-48-620-1617. E-mail address: t-taniguchi@fujiyakuhin.co.jp

Supplemental Table 1. Dosage setting of hypouricemic agents in this study

Test article	Dose in rat studies (mg/kg)	Clinical maximum dose (mg)	Clinical maximum dose (mg/kg)	Ratio
Probenecid	100	2000	33.3	3
Febuxostat	20	60	1.00	20
Benzbromarone	50	150	2.50	20
Dotinurad	1.3	4	0.0667	20

Clinical maximum doses (mg) were approved doses in Japan. Clinical maximum dose (mg/kg) was calculated as clinical maximum dose (mg) / 60 kg (as a representative body weight). Ratio was calculated as dose in rat studies (mg/kg) / clinical maximum dose (mg/kg).

Supplemental Table 2. Pharmacokinetic parameters of endogenous IS in adenine-induced acute renal failure rats

Test article	$AUC_{0\text{-}4hr}$ (ng・hr/ml)	$C_{kidney,\ 4hr}$ (ng/g tissue)	K_p
Control	37063 ± 10837	9949 ± 2652	1.3 ± 0.3
Probenecid	62289 ± 8048*	10196 ± 3074	0.6 ± 0.2**
Febuxostat	42301 ± 13029	10935 ± 2556	1.3 ± 0.3
Benzbromarone	32930 ± 5837	8091 ± 2780	1.0 ± 0.4
Dotinurad	46450 ± 18101	10888 ± 2323	1.1 ± 0.3

$AUC_{0\text{-}4hr}$; area under the curve from 0 to 4 hr, $C_{kidney,\ 4hr}$; kidney concentration at 4 hr, K_p; kidney-to-plasma partition coefficient

Data were analyzed using Phoenix WinNonlin 6.4 software (Certara, L.P., Princeton, NJ) or calculated using the following equations: K_p = kidney IS concentration at 4 hr / plasma IS concentration at 4 hr.

Each value is presented as mean ± SD of four to six animals.

*, **: $P < 0.05$, $P < 0.01$, significantly different from the control group according to Dunnett's multiple comparison test.

Supplemental Table 3. Inhibitory effects of hypouricemic agents on transporters *in vitro* and their *in vivo* risk factor as an indicator of the possible clinical inhibition of the transporters

Test article	C_{max} at clinical dose (μmol/l)	f_u	$f_u \times C_{max}$ (μmol/l)
Probenecid	79*	0.11	8.7
Febuxostat	4.1**	0.022	0.090
Benzbromarone	5.4***	0.037	0.20
Dotinurad	1.2****	0.007	0.0084

Test article	Inhibition of urate transport, IC_{50} (μmol/l)				$f_u \times C_{max}$ (μmol/l)	*In vivo* risk factor ($f_u \times C_{max}$ / IC_{50})		
	URAT1	OAT1	OAT3	ABCG2		OAT1	OAT3	ABCG2
Probenecid	165[1)]	10.9[1)]	2.37[1)]	433[1)]	8.7	0.80	3.67	0.02
Febuxostat	-	-	-	0.027[2)]	0.090	-	-	3.34
Benzbromarone	0.190[1)]	3.14[1)]	0.967[1)]	0.289[1)]	0.20	0.06	0.21	0.69
Dotinurad	0.0372[1)]	4.08[1)]	1.32[1)]	4.16[1)]	0.0084	0.00	0.01	0.00

C_{max}: maximum concentration at clinical dose; f_u: unbound fraction rate; $f_u \times C_{max}$: unbound maximum concentration; IC_{50}: half maximal inhibitory concentration.

Clinical maximum doses (mg) were approved doses in Japan. C_{max} and f_u were referenced information provided by the Pharmaceuticals and Medical Devices Agency. If an *in vivo* risk factor is above 0.1, the

drug has drug–drug interaction risk at clinical dose and it is recommended to investigate further by human drug–drug interaction study.

*: The data represents the mean concentration of q.i.d. administration, at a dose of 2 g, for 4 weeks.

**: The data represents the C_{max} of q.d. administration, at a dose of 40 mg, for 1 weeks.

***: The data represents the C_{max} of single administration, at a dose of 100 mg.

****: The data represents the C_{max} of q.d. administration, at a dose of 4 mg, for 1 weeks.

1) Inhibitory effects of probenecid, benzbromarone, and dotinurad on urate transport mediated by URAT1, OAT1, OAT3, and ABCG2 (Taniguchi, 2019)

2) The inhibitory effect of febuxostat on urate transport mediated by ABCG2 (Miyata, 2016)

Supplemental Table 4. Information of primers and antibodies

Target gene	Product code or sequence
SLC22A6 (OAT1)	TaqMan gene expression assays: Rn00568143_m1
SLC22A8 (OAT3)	TaqMan gene expression assays: Rn00580082_m1
ABCG2	TaqMan gene expression assays: Rn00710585_m1
β2-Microglobulin	Forward: 5'-TTGCCATTCAGAAAACTCCCCA-3'<br>Reverse: 5'-GCAGTTGAGGAAGTTGGGCT-3'

Antibody	Product code
Rabbit anti-OAT1 antibody	GTX64500 (GeneTex, Inc. Irvine, CA)
Rabbit ant-OAT3 antibody	KE035 (TransGenic, Inc. Fukuoka, Japan)
Rabbit anti-ABCG2 antibody	#4477 (Cell Signaling Technology, Inc. Danvers, MA)
Gout anti-rabbit IgG antibody (HRP-conjugate)	NB730-H (Novus Biologicals, LLC, Centennial, CO)

Figure Legends

Supplemental Fig. 1 Full-length blots/gels of renal expression of OAT1 (A/D), OAT3 (B/E), and ABCG2 (C/F) protein in intact and adenine-induced acute renal failure rats

The chemiluminescence in blots and the proteins in gels were detected using ChemiDoc XRS Plus (Bio-Rad Laboratories, Inc.).

Supplemental Fig. 2 Plasma concentration of hypouricemic agents in intact rats and adenine-induced acute renal failure rats

Plasma drug concentration was measured using the same samples for measuring endogenous IS and d^4-IS. Plasma samples were deproteinated with thrice volume of methanol and centrifuged at 1,000 × g for 10 min at 4°C. Drug concentrations were measured using the Alliance 2695 HPLC system (Waters Corporation. Milford, MA).

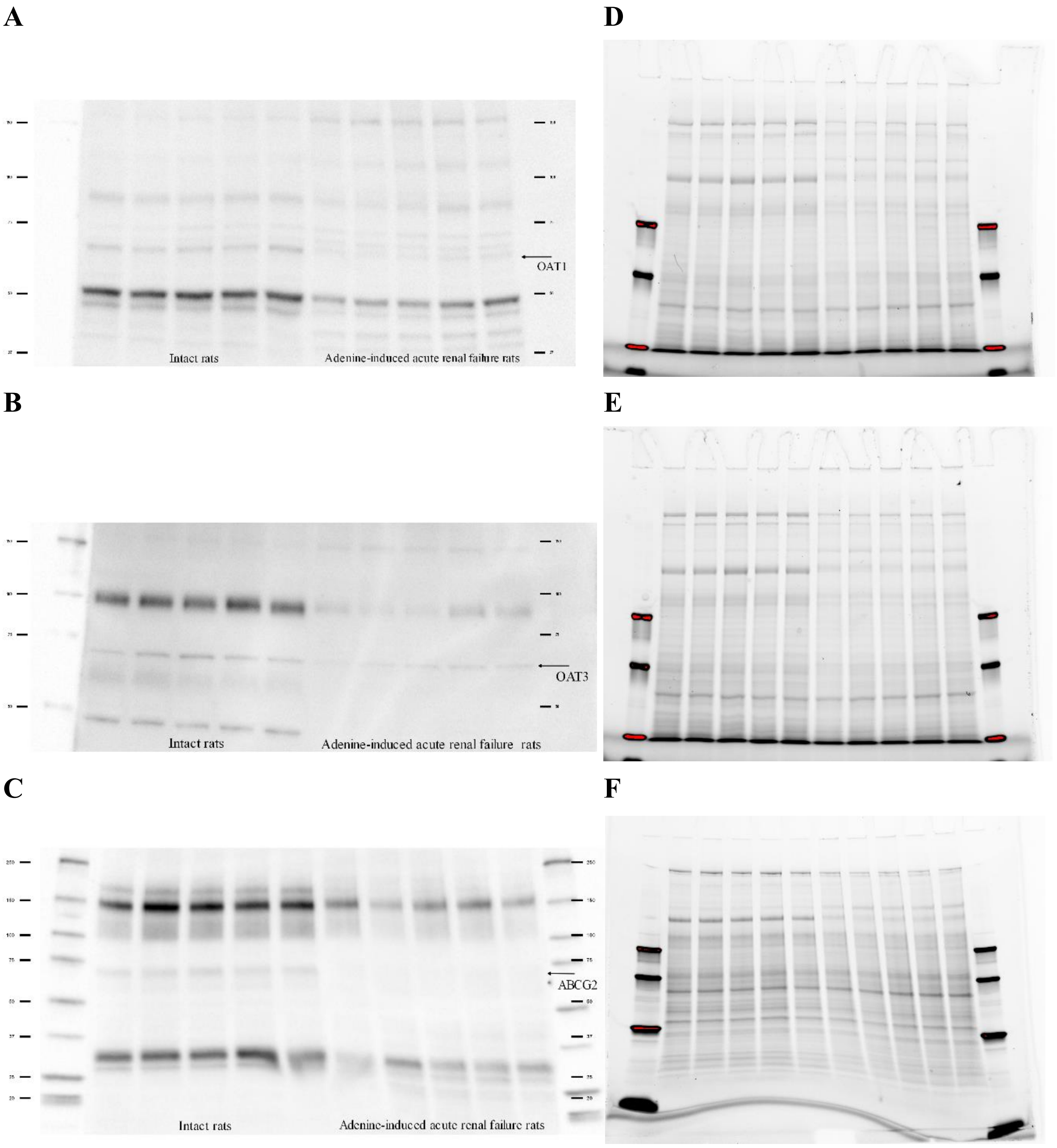

Supplemental Fig. 1 Full-length blots/gels of renal expression of OAT1 (A/D), OAT3 (B/E), and ABCG2 (C/F) protein in intact and adenine-induced acute renal failure rats

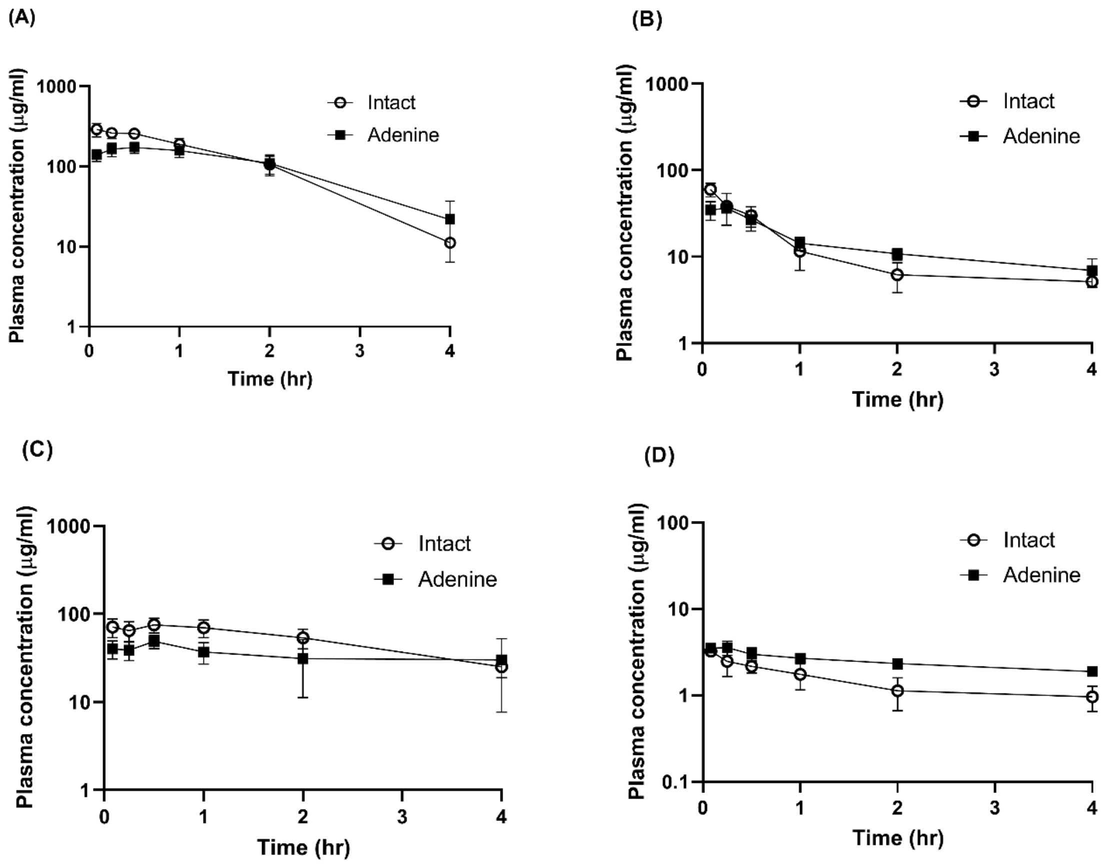

Supplemental Fig. 2 Plasma concentration of hypouricemic agents in intact rats and adenine-induced acute renal failure rats
